# Prospective trial examining safety and efficacy of microcurrent stimulation for the treatment of sinus pain and congestion

**DOI:** 10.1186/s42234-019-0035-x

**Published:** 2019-11-20

**Authors:** Alan B. Goldsobel, Niveditha Prabhakar, Blake T. Gurfein

**Affiliations:** 1 Allergy and Asthma Associates of Santa Clara Valley Research Center, San Jose, CA USA; 2 Stanford University School of Medicine, Stanford, CA USA; 3 University of California San Francisco, San Francisco, CA USA; 4 Tivic Health Systems, Inc., 750 Menlo Ave #200, Menlo Park, CA 94025 USA

**Keywords:** Transcutaneous electrical nerve stimulation, Microcurrent, Facial pain, Sinus pain, Rhinologic facial pain, Congestion, Nasal congestion, Allergic rhinitis, Cranial nerve, Trigeminal nerve

## Abstract

**Background:**

Inflammation and swelling of the sinus and nasal mucosa are commonly caused by viral infection, bacterial infection, or exposure to allergens and irritants. Sinonasal inflammation can cause symptoms of nasal congestion, facial pressure, and rhinogenic facial pain or “sinus pain”. A previous randomized controlled study demonstrated that acute treatment with non-invasive periorbital microcurrent stimulation resulted in a rapid and clinically meaningful reduction in self-report of sinus pain that significantly outperformed sham control treatment. Here, we assessed the acute durability of microcurrent pain relief and longitudinal effects of 4 weeks of daily microcurrent treatment in patients presenting with sinus pain.

**Methods:**

Thirty subjects with moderate facial pain (numeric rating scale ≥5) attributed to self-reported sinonasal disease were enrolled in a single-arm, prospective interventional study. At enrollment, subjects were given a microcurrent treatment device and written instructions and self-administered the device to the bilateral periorbital regions for 5 mins. Subjects were instructed to treat themselves at home once daily and up to four times daily as needed for 4 weeks. Pain was measured both acutely and weekly during the 4 weeks of treatment using the numeric rating scale. Congestion and medication use data were collected weekly using the Congestion Quantifier 7 (CQ7) and medication diary, respectively.

**Results:**

Thirty patients were enrolled and completed the study. Microcurrent therapy rapidly reduced post-treatment numeric rating scale for pain by − 1.2 at 10 mins (*p* = 0.0076), − 1.6 at 1 hr (*p* = 0.0007), − 1.9 at 2 hrs (*p* < 0.0001), − 2.1 at 4 hrs (*p* < 0.0001), and − 2.1 at 6 hrs (*p* < 0.0001). With daily microcurrent treatment, numeric rating scale for pain was reduced over 4 weeks by − 1.3 (− 20.1%) after 1 week (*p* = 0.0018), − 2.1 (− 32.1%) after 2 weeks (*p* < 0.0001), − 2.4 (− 36.6%) after 3 weeks (*p* < 0.0001) and − 2.9 (− 43.3%) after 4 weeks (*p* < 0.0001). For subjects who enrolled with moderate or worse congestion, mean congestion scores (CQ7) were reduced by − 4.2 (− 22.0%) after 1 week (*p* < 0.0001), − 5.8 (− 33.0%) after 2 weeks (*p* < 0.0001), − 7.2 (− 37.4%) after 3 weeks (*p* < 0.0001) and − 8.6 (− 44.3%) after 4 weeks (*p* < 0.0001) of microcurrent treatment.

**Conclusion:**

Self-administered periorbital microcurrent treatment given at home was efficacious in significantly reducing moderate sinus pain for up to 6 hrs and significantly reducing moderate pain and congestion over 4 weeks of daily use. Microcurrent therapy was found to be safe with only minor side effects that resolved without intervention.

**Trial registration:**

ClinicalTrials.gov, NCT03888274. Registered 25 March 2019. Retroactively registered, https://clinicaltrials.gov/ct2/show/NCT03888274.

## Introduction

Inflammation of the sinuses and nasal mucosa results in symptoms such as thick nasal mucus, tissue swelling, nasal congestion or obstruction, and pain and pressure in the face (Rosenfeld et al. 2015; Seidman et al. 2015). Patients with rhinologic facial pain, or “sinus pain”, typically describe the quality of the pain as pressure (Cady and Schreiber 2004). Sinonasal inflammation can be caused by infection, allergies, air pollution and other irritants, or structural problems in the nasal passages. Sinonasal inflammation is a common condition that affects all age groups and women are more often affected than men (Blackwell et al. 2015). Rhinosinusitis and allergic rhinitis are the commonest causes of sinonasal inflammation and impact approximately 12% and 30% of the population, respectively (Blackwell et al. 2015; Meltzer and Bukstein 2011; Salo et al. 2011). Treatment of rhinosinusitis and allergic rhinitis alone results in billions of dollars in healthcare costs each year (Meltzer and Bukstein 2011; Smith et al. 2015).

Facial pain of sinonasal origin is treated with analgesic and anti-inflammatory agents, including ibuprofen, acetaminophen, and oral or intranasal corticosteroids (Rosenfeld et al. 2015). Nasal congestion is treated with nasal irrigation, intranasal glucocorticoids (fluticasone propionate, mometasone furoate) oral decongestants (phenylephrine, pseudoephedrine), and intranasal decongestants (oxymetazoline) (Corey et al. 2000). These drugs have low to moderate efficacy for treating pain and congestion and present challenges driven by high cost, potential for dependency, and tolerability. For example, medications for treating pain can cause gastrointestinal discomfort and bleeding and medications for nasal congestion have side effects including nervousness, insomnia, epistaxis, and rhinitis medicamentosa, among others (Jin 2015; Ramey et al. 2006). Novel well-tolerated therapies that can be used by the at-large population affected by sinus pain and congestion would provide significant clinical and economic benefit.

Maul and colleagues previously conducted a randomized controlled double-blinded trial in which a handheld transcutaneous microcurrent-emitting device was assessed as a treatment for patients with sinus pain (Maul et al. 2019). Enrolled patients with sinus pain (*N* = 71), originating predominately from rhinosinusitis and allergic rhinitis, self-administered microcurrent or sham treatment to the bilateral periorbital regions for 5 mins and visual analog scale for pain was collected before and 10 mins after treatment. The study results demonstrated that bioelectronic microcurrent treatment rapidly induced a clinically meaningful reduction in sinus pain (− 29.6%) that was significantly greater than the pain reduction observed in sham-treated patients.

The current study is a prospective, single-arm, open label study intended to determine the durability of the analgesic effect of microcurrent therapy and generate longitudinal data on pain, congestion, medication use, and feasibility during 4 weeks of at-home microcurrent therapy. Enrolled subjects presenting with sinus pain self-administered the microcurrent device to the bilateral periorbital areas for 5 mins during the first study visit and then took the device home with instructions to apply the treatment once daily and up to four times daily as needed for 4 weeks. Data from validated instruments for quantifying pain and congestion and a medication diary were collected weekly for the duration of the study.

## Methods

### Study subjects

Thirty study subjects, 21 females and 9 males, with self-reported rhinologic facial pain or “sinus pain” were recruited from the Allergy and Asthma Associates of Santa Clara Valley Research Center in San Jose, CA and the surrounding community. Study subject etiologies of sinus pain included allergic rhinitis, nasal polyps, or undiagnosed/unknown etiologies. Inclusion criteria were: 18–71 years of age (inclusive), symptoms of sinus pain or facial pain in the forehead, periorbital, facial, or nasal region, pain score ≥ 5 (Numeric Rating Scale 0–10), frequency of sinus/facial pain at least twice weekly for 1 month or more, able to read and understand English, agree to participate in the study, able and willing to provide informed consent. Exclusion criteria were: do not meet inclusion criteria, currently taking or recently taken any oral steroid medications in the last 90 days, sinus surgery in previous 90 days, history of chronic migraine (≥ 15 headache days per month), pain location in the vertex, occiput, or temporal region of the skull or in mandibular region, purulent rhinorrhea, current dental infection, cranial nerve pathology (trigeminal neuralgia, facial nerve paralysis, etc.), primary pain disorder (fibromyalgia, chronic regional pain syndrome, etc.), implanted electrostimulation devices (pacemaker, a deep brain stimulator, or a cochlear implant).

### Study design

This study was conducted after written approval of the study protocol and informed consent by an institutional review board. All subjects were enrolled in a single-arm, prospective interventional study. Eligible subjects read and provided written informed consent at the enrollment study visit. Validated questionnaires were used to quantify sinonasal symptoms including pain (numeric rating scale) and congestion (Congestion Quantifier 7) (Stull et al. 2007). Data on medication use was collected via medication diary. Subjects used the microcurrent device for 5 mins during the study visit and then took the device home with them for 4 weeks with instructions to use the device once daily and up to four times daily as needed, with each treatment lasting 5 mins. No further instructions were given to the study subjects regarding what time of day to use the device or the frequency that the device should be used. Acute pain relief data was collected after the first study visit treatment (10 min, 1 h, 2 h, 4 h, 6 h after treatment) and data on pain, congestion, and medication use was collected weekly for 4 weeks. Once per week, subjects completed a pain data sheet that contained a numeric rating scale for pain for their current pain and their best, worst, and average pain over the previous week. The Congestion Quantifier 7 (CQ7) is a validated instrument that measures the severity of sinonasal congestion symptoms over the previous week and was completed by study subjects once weekly (Stull et al. 2007). The CQ7 quantifies the frequency of symptoms and problems such as nasal stuffiness, blockage, and congestion, pressure and pain in the face, breathing through the mouth, difficulty clearing the nose, impact on school or work, congestion upon waking, and negative impact on sleep. At the end of the four-week study period, a questionnaire was administered to each subject to collect data on device usability, study subject satisfaction, and safety. Daily and weekly text message reminders were sent to each study subject to remind them to use the device daily and to complete weekly questionnaires. To facilitate a real-world pragmatic trial, subjects were allowed to use medication for sinus pain during the study with the exception of oral steroids. Study visits took place at enrollment and at 4 weeks.

### Intervention

**Fig. 1:**
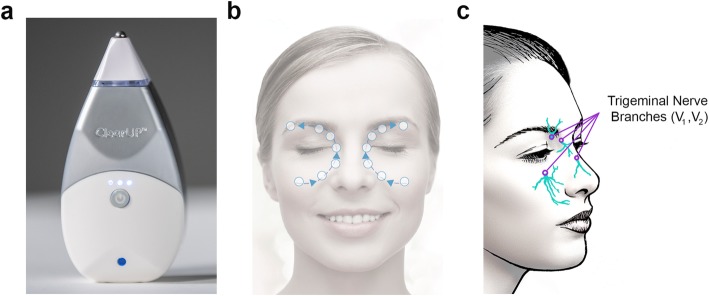
Microcurrent device and treatment path. The microcurrent device used in this study has a stimulation electrode at the tip and a return electrode built into the housing, comprising a monopolar design (**a**). Subjects were instructed to self-administer the device and follow a treatment path around the bilateral periorbital areas along the cheek, nose, and brow ridge (**b**). Subcutaneous fibers of trigeminal nerve branches V_1_ (ophthalmic nerve) and V_2_ (maxillary nerve) are targets of microcurrent stimulation (**c**)

The study device, ClearUP™ Sinus Pain Relief (Tivic Health Systems, Inc., Menlo Park, CA), is a non-invasive, handheld stimulator that emits microcurrent through its conductive tip and has a current return path through the conductive housing of the handset (Fig. 1a). The device emits biphasic current at extremely low frequency (3–30 Hz) and delivers a maximum current of 2.4–2.52 milliamps at 500 Ω. The device is FDA-cleared for the temporary relief of sinus pain associated with allergic rhinitis. Treatment with the study device involves putting the stimulation electrode in contact with the skin and gliding the device in a confined, repetitive “H” pattern above and below the bilateral orbits for 5 min to span between the bilateral supraorbital and infraorbital nerve regions and across the nasal dorsum (Fig. 1b). The device uses a proprietary algorithm to detect treatment points, areas where there is a density of subcutaneous nerve fibers including ophthalmic (V_1_) and maxillary (V_2_) branches of the trigeminal nerve. The device emits microampere alternating current in these regions (Fig. 1c). When a treatment point is detected, the device emits haptic vibration to let the user know to hold the device in place temporarily for 7 seconds while the stimulation is applied to the treatment point. The device constantly emits current during the 5 min treatment period. All study subjects self-administered the microcurrent device treatment during the enrollment study visit for 5 mins. Additionally, all subjects were given a device to take home and instructed to use the device once daily and up to four times per day as needed.

### Statistical analysis

**Fig. 2:**
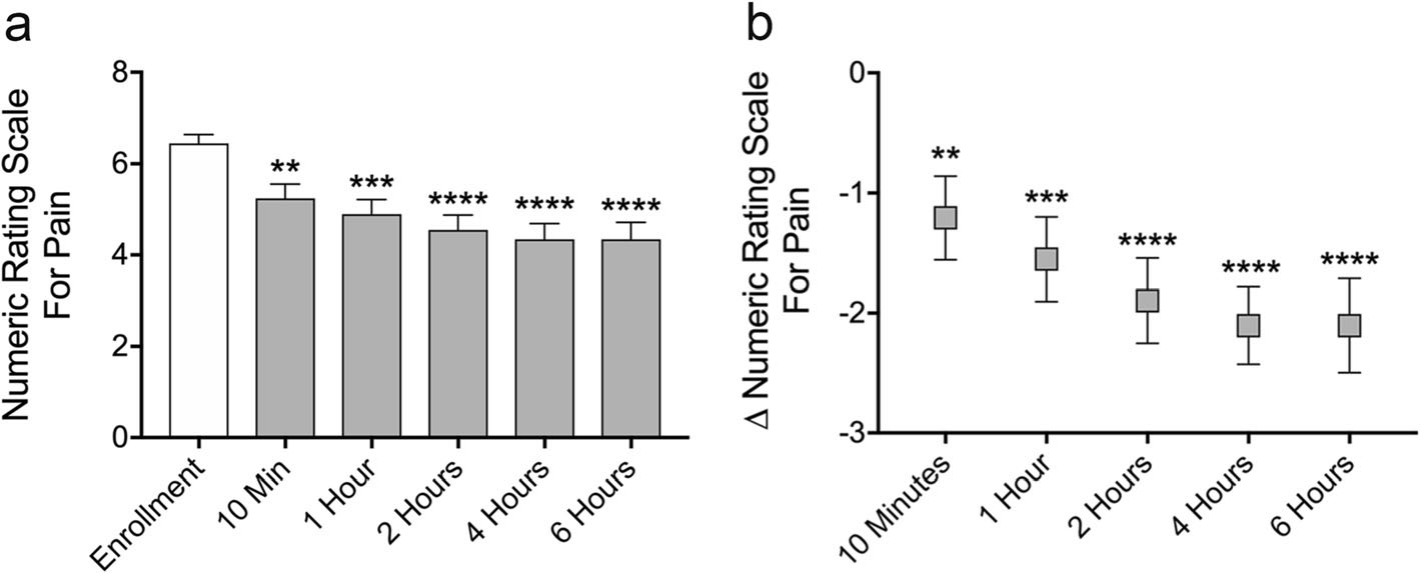
Microcurrent treatment rapidly reduces sinus pain for up to 6 h. Thirty subjects with sinus pain self-administered microcurrent treatment to the bilateral periorbital areas for 5 mins and numeric rating scale for pain scores were recorded before and after treatment. Mean numeric rating scale scores were significantly reduced from 6.5 at pretreatment to 4.3 at 6 h post-treatment (**a**). Mean difference in pain score from before treatment peaked at − 2.1 numeric rating scale points at 4 hrs (**b**). Data represented as mean ± SEM. **p < 0.01, ***p < 0.001, ****p < 0.0001, repeated measures one-way ANOVA with Dunnett correction for multiple comparisons

**Fig. 3:**
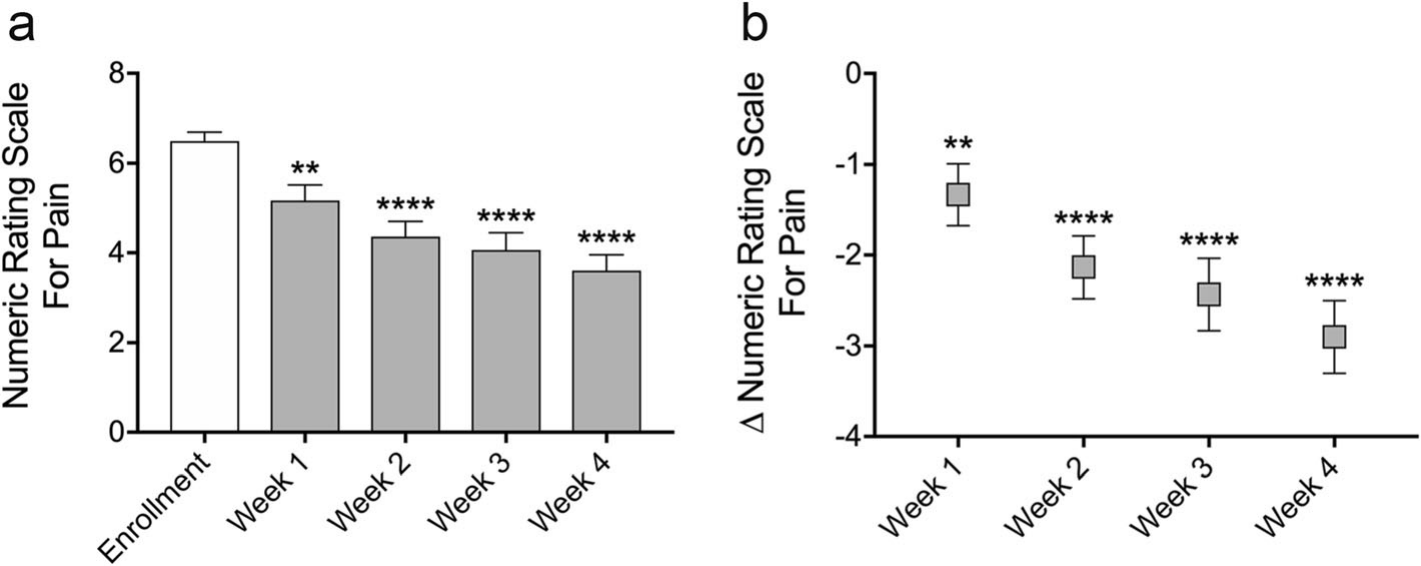
Daily microcurrent treatment reduces current sinus pain score over 4 weeks. Subjects with sinus pain self-administered microcurrent treatment at home once per day and up to four times per day as needed for 4 weeks. Numeric rating scale for pain was collected weekly. Compared to before treatment, mean current numeric rating scale for pain scores were significantly reduced at all time points measured (**a**). Mean difference in current pain score from before treatment peaked at − 2.9 (− 43.3%) numeric rating scale points at week four (**b**). Data represented as mean ± SEM. **p < 0.01, ****p < 0.0001, repeated measures one-way ANOVA with Dunnett correction for multiple comparisons

**Fig. 4:**
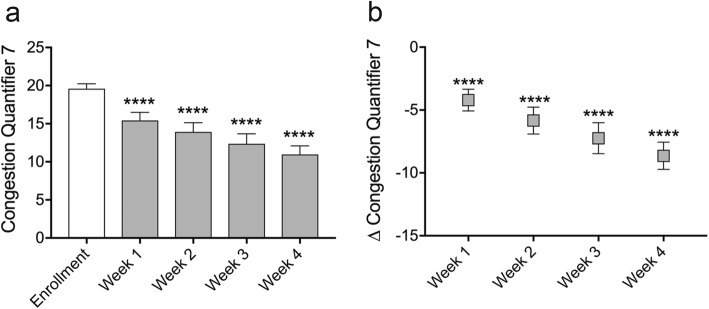
Daily microcurrent treatment reduces sinonasal congestion over 4 weeks. Sinonasal congestion was assessed weekly using the Congestion Quanitfier 7 (CQ7) instrument. Subjects that enrolled with moderate or worse congestion (CQ7 > 15, *N* = 25) exhibited significant reductions in congestion symptoms, compared to pretreatment, at all time points measured (a). Mean difference in CQ7 score from before treatment peaked at − 8.6 (− 44.3%) points at week four (b). Data represented as mean ± SEM. ****p < 0.0001, paired, two-sided t-test

**Fig. 5:**
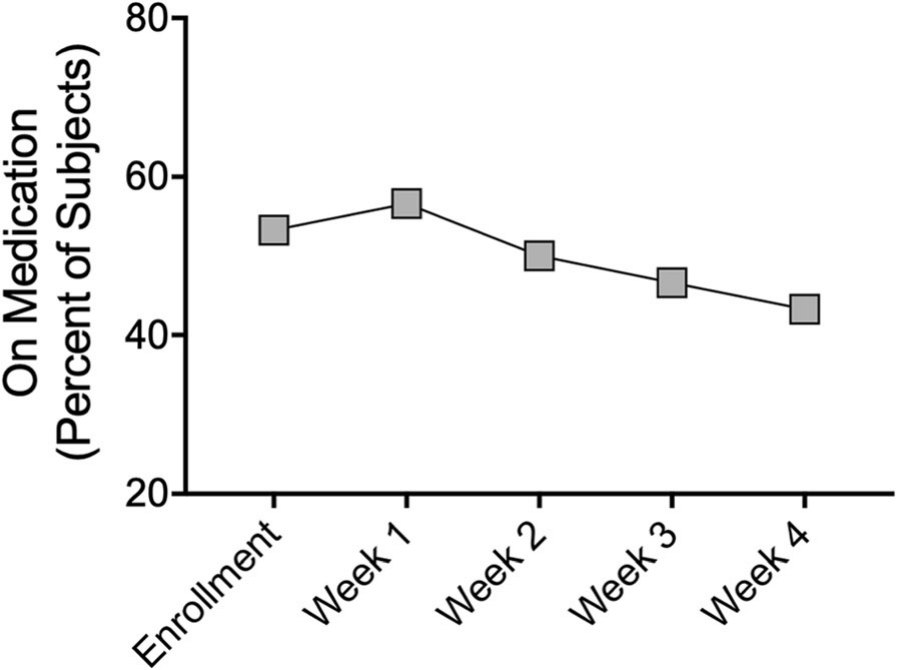
Concurrent medication use during 4 weeks of microcurrent treatment. At enrollment and weeks one through four of treatment, subjects completed a medication diary detailing the classes of medication they used in the previous week including: pain, nasal decongestant spray, nasal steroid spray, decongestant in pill form, antihistamine, or prescription antibiotics. A modest trend towards decreased reliance on medication was observed from enrollment to week four, however these results were not statistically significant. Data represented as percentage of subjects on one or more medications. Chi-square test for trend

Repeated measures one-way ANOVA with Dunnett correction for multiple comparisons was conducted for Figs. 2 and 3. Paired two-sided t-tests were conducted for Fig. 4. A Chi-square test for trend was conducted for Fig. 5. Statistical analysis was carried out using Graphad Prism 7.0d.

## Results

### Microcurrent treatment rapidly reduces sinus pain for up to 6 h

In this study, thirty patients presenting with rhinologic facial pain (“sinus pain”) were recruited from a tertiary allergy clinic. To be eligible for enrollment, subjects were required to have experienced sinus pain at least twice weekly for 4 weeks or more at the time of screening, indicating persistent symptomatology. Baseline subject characteristics are detailed in Table 1. The etiologies of sinus pain reported by study subjects were allergic rhinitis (36.7%), polyps (6.7%), and unknown or undiagnosed (56.6%). To assess the acute analgesic effects of microcurrent treatment, subjects self-administered a handheld microcurrent emitting device to the bilateral periorbital areas for 5 mins in the Research Center. Numeric rating scale for pain was collected before treatment and 10 mins, 1 hr, 2 hrs, 4 hrs, and 6 hrs after treatment. Before microcurrent treatment, subjects reported a mean numeric rating scale score of 6.5. After treatment mean pain scores were 5.2 at 10 mins (*p* = 0.0076), 4.9 at 1 hr (*p* = 0.0007), 4.6 at 2 hrs (*p* < 0.0001), 4.3 at 4 hrs (*p* < 0.0001), and 4.3 at 6 hrs (*p* < 0.0001) (Fig. 2a). The mean difference in pain score from before treatment was − 1.2 (95% Confidence Interval (CI_95_) -0.3 to − 2.1, *p* = 0.0076) at 10 mins, − 1.6 (CI_95_–0.6 to − 2.5, *p* = 0.0007) at 1 hr, − 1.9 (CI_95_–0.9 to − 2.8, *p* < 0.0001) at 2 hrs, − 2.1 (CI_95_–1.2 to − 3.0, *p* < 0.0001) at 4 hrs, and − 2.1 (CI_95_–1.1 to − 3.1, *p* < 0.0001) at 6 hrs (Fig. 2b). Repeated measures analysis indicated that the decrease in pain over the six-hour observation period was highly statistically significant (*p* < 0.0001). Previous studies have found that a 1.3 point reduction in pain on a 10 point scale is the minimum clinically meaningful difference for acute pain (Gallagher et al. 2001; Todd et al. 1996). After the first 5 min treatment self-administered by the subjects the proportion of subjects that reported a clinically meaningful change in pain was 31.0% (9/29) at 10 mins, 41.4% (12/29) at 1 hr, 55.2% (16/29) at 2 hrs, 55.2% (16/29) at 4 hrs, and 55.2% (16/29) at 6 hrs.

**Table 1:** Subject characteristics at baseline

	Mean	S.D.	Min	Max
Current Pain (Numeric Rating Scale)	6.5	1.1	5.0	9.0
Composite Pain (Numeric Rating Scale)	24.7	4.0	19.0	32.0
Number of Concurrent Medications	0.8	1.0	0.0	4.0

Composite pain is calculated as the sum of current pain and best, worst, and average pain over the previous week. S.D., standard deviation

### Daily microcurrent treatment reduces sinus pain over four weeks

At enrollment, before treatment, the mean numeric rating scale score for current pain was 6.5. Mean current pain scores were 5.2 (− 20.1%) after 1 week (*p* = 0.0018), 4.4 (− 32.1%) after 2 weeks (*p* < 0.0001), 4.1 (− 36.6%) after 3 weeks (*p* < 0.0001) and 3.6 (− 43.3%) after 4 weeks (*p* < 0.0001) of microcurrent treatment (Fig. 3a, Table 2). When compared with pre-treatment scores at enrollment, the mean difference in current pain score was − 1.3 (CI_95_–0.5 to − 2.2, *p* = 0.0018) at week one, − 2.1 (CI_95_–1.2 to − 3.1, *p* < 0.0001) at week two, − 2.4 (CI_95_–1.4 to − 3.5, *p* < 0.0001) at week three, and − 2.9 (CI_95_–1.9 to − 4.0, *p* < 0.0001) at week four (Fig. 3b). Repeated measures analysis indicated that the decrease in current pain over the four-week study was highly statistically significant (*p* < 0.0001). The proportion of subjects that reported a clinically meaningful change in current pain was 46.7% (14/30) at week one, 56.7% (17/30) at week two, 66.7% (20/30) at week three, and 70.0% (21/30) at week four. A composite pain score was also calculated as the sum of the numeric rating scale for current pain and best, worst, and average pain over the previous week. At enrollment, before treatment, the mean composite numeric rating scale score for pain was 24.7. Mean composite pain scores were 18.7 after 1 week (*p* < 0.0001), 17.6 after 2 weeks (*p* < 0.0001), 17.1 after 3 weeks (*p* = 0.0001) and 14.6 after 4 weeks (*p* < 0.0001) of microcurrent treatment (Additional file 1: Figure S1a). When compared with pre-treatment scores at enrollment, the mean difference in composite pain score was − 6.1 (CI_95_–3.4 to − 8.7, *p* < 0.0001) at week one, − 7.1 (CI_95_–4.0 to − 10.3, *p* < 0.0001) at week two, − 7.6 (CI_95_–3.6 to − 11.6, *p* = 0.0001) at week three, and − 10.1 (CI_95_–6.3 to − 13.9, *p* < 0.0001) at week four (Additional file 1: Figure S1b).

**Table 2:** Percentage change in sinus symptom severity

	Week 1	Week 2	Week 3	Week 4
Δ Numeric Rating Scale for Pain	−20.1%	−32.1%	−36.6%	−43.3%
Δ Congestion Quantifier 7 Score	−22.0%	−33.0%	−37.4%	−44.3%

Percentage change in symptom severity when compared with enrollment scores

### Daily microcurrent treatment reduces sinonasal congestion over four weeks

The CQ7 was administered at enrollment, before treatment, and then weekly for the four-week duration of the study. Enrolled patients with sinus pain of 5 or greater on the numeric rating scale had a high degree of sinonasal congestion comorbidity, with 25 of 30 subjects (83.3%) reporting moderate or worse (CQ7 > 15) congestion symptoms at enrollment. In this population, at enrollment, before treatment, the mean CQ7 score was 19.6. Mean CQ7 scores were 15.4 (− 22.0%) after 1 week (*p* < 0.0001), 13.9 (− 33.0%) after 2 weeks (*p* < 0.0001), 12.4 (− 37.4%) after 3 weeks (*p* < 0.0001) and 11.0 (− 44.3%) after 4 weeks (*p* < 0.0001) of microcurrent treatment (Fig. 4a, Table 2). When compared with pre-treatment scores at enrollment, the mean difference in CQ7 score was − 4.2 (CI_95_–2.4 to − 6.0, *p* < 0.0001) at week one, − 5.8 (CI_95_–3.6 to − 8.0, *p* < 0.0001) at week two, − 7.2 (CI_95_–4.7 to − 9.8, *p* < 0.0001) at week three, and − 8.6 (CI_95_–6.4 to − 10.9, *p* < 0.0001) at week four (Fig. 4b). Previous studies have concluded that a CQ7 score reduction of three points or greater indicates a clinically meaningful improvement in symptom severity has occurred (Stull et al. 2008).

### Concurrent medication use during 4 weeks of microcurrent treatment

To facilitate a pragmatic study design, subjects were permitted to use medications for sinus pain during the trial, with the exception of oral corticosteroids, which were excluded. At enrollment and weeks one through four of treatment, subjects completed a medication diary detailing the classes of medication they used in the previous week. Medication classes included: pain, nasal decongestant spray, nasal steroid spray, oral decongestants, oral antihistamines, or prescription antibiotics. The mean number of medications reported were 0.8 at enrollment, 0.83 at week one, 0.73 at week two, 0.70 at week three, and 0.63 at week 4. Differences in mean number of medications used each week compared with enrollment were not statistically significant. At enrollment, 16 subjects (53.3%) had taken one or more medications in the previous week. The number of subjects on one or more medications was 17 (56.6%) during week one, 15 (50.0%) during week two, 14 (46.6%) during week three, and 13 (43.3%) during week four (Fig. 5). The modest trend towards reduced reliance on medication was not statistically significant (*p* = 0.2987).

### Safety and user experience

At the conclusion of the study, subjects completed a questionnaire in which they were asked to report any potential adverse effects and provide qualitative feedback on their experience using the microcurrent treatment over 4 weeks. Subjects reported they experienced mild transient erythema (*N* = 2, 6.6%), headache (*N* = 1, 3.3%), and eyelid twitch (N = 1, 3.3%). All reports of side effects were considered minor and resolved without intervention. 76.6% of subjects reported that they were satisfied with home use of the microcurrent device and that they would recommend it to other patients with sinus symptoms.

## Discussion

A previously published randomized, sham-controlled, double blinded study demonstrated that active microcurrent therapy reduced sinus pain acutely and the magnitude of the analgesic effect was significantly greater than that observed in sham-treated patients (Maul et al. 2019). In the current study, the acute durability of microcurrent-induced analgesia was measured in addition to longitudinal assessment of sinus pain, nasal congestion, medication use, safety, and user experience during 4 weeks of regular microcurrent therapy. After a five-minute microcurrent treatment, the mean reduction in numeric scale for pain was greatest at 4 hrs and pain relief lasted up to 6 hrs, the longest time point measured. Future trials will examine acute time points longer than 6 hrs after treatment to further define the therapeutic response curve. Weekly assessment of sinus pain using the numeric rating scale demonstrated that daily microcurrent treatment was associated with statistically significant reductions in pain, compared with before treatment, at all time points measured. Notably, the analgesic effect increased over the 4 weeks of treatment, peaking at − 43.3% at week four. Similarly, congestion severity, as measured by the CQ7 instrument, was significantly reduced at all time points measured, peaking at − 44.3% at week four. Microcurrent therapy was associated with a modest non-significant trend towards reduced medication use over the 4 week study. Importantly, at-home microcurrent device use was found to be feasible and very well tolerated by study subjects, with few occurrences of minor side effects.

While few studies have been conducted to measure the magnitude of sinus pain relief achieved by over-the-counter analgesics such as ibuprofen and acetaminophen, the durability of pain relief provided by those agents is reported to be four to 6 hrs (Ong et al. 2007). Comparably, this study demonstrated that microcurrent treatment analgesia lasted up to 6 hrs after the first treatment. Research on the efficacy of decongestant medications has shown that phenylephrine can reduce nasal congestion severity by − 7.1% to − 25% and regulated formulations of pseudoephedrine can reduce congestion symptoms by approximately − 21.7% (Horak et al. 2009; Meltzer et al. 2015). Fluticasone propionate, one of the more efficacious drug therapies available for nasal congestion, has been reported to reduce congestion symptoms by − 33.6% to − 47% depending on the study (Nathan et al. 2005; Ratner et al. 2002). Microcurrent treatment decongestant effects ranged from − 22.0% at week one to − 44.3% at week four, which is similar to or greater than the decongestant effects observed in many over-the-counter oral and intranasal drug studies and with a more attractive safety profile.

Transcutaneous electrical nerve stimulation (TENS) devices have been used to treat a variety of types of chronic pain. While, TENS treatment is not indicated for use on the head or neck, a previous study found that conventional TENS therapy significantly reduced facial myalgia pain compared to control treatment (De Giorgi et al. 2017). The microcurrent device studied in this trial passes current transcutaneously, as TENS devices do, however the waveform frequency and amplitude and the mode of current delivery were developed specifically to treat sinonasal symptoms safely, effectively, and with a high degree of user comfort. Importantly, this is the first report of a bioelectronic device capable of generating decongestant effects in addition to the analgesia normally associated with neuromodulation management of pain.

The microcurrent device studied in this trial has two unique design features. First, the device uses a proprietary algorithm to identify treatment points. When the device identifies a treatment point, haptic vibration indicates to the user that the device should be held stationary. These treatment points are areas where the cutaneous resistance to current is low, allowing for efficient delivery of stimulation. Previous work has shown that dermal regions that are directly above subcutaneous nerve tissue have lower resistance and higher capacitance than dermal regions that do not contain subcutaneous nerves (Prokhorov et al. 2002). Second, the device has a monopolar design in which current passes through the stimulation electrode at the tip of the device and returns via the conductive housing of the device which serves as the return electrode. The monopolar design facilitates a greater depth of penetration by creating a current path from the stimulation electrode into the face and then back to the housing of the device via the user’s arm.

Microcurrent stimulation of the face is understood to exert therapeutic effects via two principal pathways. First, delivery of electrical microcurrent in the periorbital regions stimulates subcutaneous fibers of the trigeminal nerve (V_1_, ophthalmic nerve and V_2_, maxillary nerve), which are responsible for relaying sensory information for the face, nose, and sinuses to the brain (Huff and Daly 2019). Neuromodulation of the trigeminal nerve pathway alleviates sensations of pain and pressure (Hansson and Ekblom 1983; Slavin et al. 2006). Second, there is pervasive sympathetic innervation of the blood vessels that supply the sinus and nasal mucosa (Naclerio et al. 2010; Sahin-Yilmaz and Naclerio 2011). This sympathetic innervation by postganglionic nerve fibers facilitates vasoconstriction by the release of norepinephrine and subsequent smooth muscle contraction (Fischer et al. 1993). Electrical stimulation of sympathetic nerve fibers can promote release of norepinephrine and vasoconstriction as evidenced by several studies investigating frequency ranges similar to that of the microcurrent study device (Franco et al. 2014; Malm 1973; Mandel et al. 2013). Vasoconstriction of arterioles and venous vessels, in the context of sinonasal inflammation, results in smaller vessel diameter, reduced edema and extravasation of inflammatory immune cells, as well as less nasal resistance to air flow, all of which can contribute to reduced symptom m severity and decongestant effects (Naclerio et al. 2010).

A key limitation of the study design was that it did not include a sham-treated control group and, thus, quantification of the contribution of placebo is not possible. While this design limits the interpretation of the results, previous double-blinded randomized controlled clinical trial, using the same study device, demonstrated that a single application of active microcurrent treatment resulted in a mean reduction in acute sinus pain of 29.6% and the magnitude of pain reduction was significantly greater than that observed in sham-treated control subjects (Maul et al. 2019). In that trial, the sham device appeared identical to the active device and emitted haptic feedback, however it did not deliver alternating current to the subject. These results indicate that the analgesic effect observed from microcurrent treatment are significantly greater than placebo alone. A sham control group in the current study was considered and would have presented several challenges. The most critical challenge for a longitudinal study investigating an electrical stimulation device is maintaining a sufficient blinding index in sham-treated control subjects. Due to the four-week duration of the study it is anticipated that most subjects randomized to sham treatment would be unblinded by the end of the trial, undermining the usefulness of the comparison data. In future clinical studies, implementation of alternative control groups such as waitlist, usual care, or even placebo drug will be explored.

Another limitation of the study was that some of the recruited subjects had not been previously diagnosed regarding the etiology of their sinus symptoms. Given our epidemiologic knowledge of the commonest causes of sinonasal pain, congestion, and inflammation, it is likely that these subjects were experiencing either allergic or non-allergic rhinitis or rhinosinusitis, though the diagnoses remain unconfirmed (Blackwell et al. 2015; Meltzer and Bukstein 2011; Salo et al. 2011). While better characterization of the patient population would be helpful, it is encouraging that a heterogenous patient population presenting with sinus pain was observed to have large clinically meaningful improvements in pain and congestion symptoms. Furthermore, the biological underpinnings of sinus pain and nasal congestion, such as edema, activation of afferent trigeminal nerve pathways, and mucosal inflammation, are understood to occur across conditions such as allergic rhinitis and rhinosinusitis and, thus the efficacy of microcurrent treatment would not be expected to differ.

Lastly, as is the case with many pragmatic at-home clinical studies, it cannot be definitively demonstrated that the study device was used as intended by the study protocol. While text message reminders are helpful tools to ensure compliance, the frequency and duration of device use and the anatomical locations stimulated cannot be confirmed, with the exception of the treatments that occurred during the enrollment study visit. While this is a weakness of home-use interventional studies, a previous trial with the study device demonstrated that subjects were able to read the instructions for use and self-administer the device resulting in safe and effective clinical benefit without guidance from study staff or clinicians (Maul et al. 2019).

## Conclusion

This study demonstrated that non-invasive bioelectronic microcurrent therapy acutely reduced rhinologic facial pain for up to 6 h and, with regular use, alleviated the severity of sinus pain and nasal congestion over 4 weeks. The magnitude of the therapeutic effect observed was comparable to or greater than that of widely used over-the counter drugs. Microcurrent treatment was very well tolerated and caused few occurrences of minor side effects. These results suggest that microcurrent treatment is safe and effective provides an important non-opioid, non-pharmaceutical treatment option for the large population of patients that suffer from sinonasal pain and congestion.

## Supplementary information

**Additional file 1: Figure S1.** Daily microcurrent treatment reduces composite sinus pain score over 4 weeks. Numeric rating scale for pain was collected weekly including current pain and best, worst, and average pain over the previous week. A composite pain score was calculated by adding all four pain scales at each time point measured. Compared to before treatment, mean composite numeric rating scale for pain scores were significantly reduced at all time points measured (a). Mean difference in current pain score from before treatment peaked at − 10.1 points at week four (b). Data represented as mean ± SEM. ***p < 0.001, ****p < 0.0001, repeated measures one-way ANOVA with Dunnett correction for multiple comparisons.

## Data Availability

Please contact authors for data requests.

## Abbreviations

ANOVA: Analysis of Variance
CI_95_: 95% confidence interval
CQ7: Congestion Quantifier 7
FDA: Food and Drug Administration
S.D.: Standard Deviation
SEM: Standard error of the mean
