# Correction to: Prospective trial examining safety and efficacy of microcurrent stimulation for the treatment of sinus pain and congestion

**DOI:** 10.1186/s42234-020-0039-6

**Published:** 2020-01-30

**Authors:** Alan B. Goldsobel, Niveditha Prabhakar, Blake T. Gurfein

**Affiliations:** 1 Allergy and Asthma Associates of Santa Clara Valley Research Center, San Jose, CA USA; 2 Stanford University School of Medicine, Stanford, CA USA; 3 University of California San Francisco, San Francisco, CA USA; 4 Tivic Health Systems, Inc., 750 Menlo Ave #200, Menlo Park, CA 94025 USA

**Correction to: Bioelectronic Med (2019) 5:18**

**https://doi.org/10.1186/s42234-019-0035-x**

The original version of this article (Goldsobel et al. 2019), published on 20 November 2019, contained incorrect data. In this Correction the affected parts of the article are shown.

Figure 4 contained incorrect data. The correct number is marked bold and shown below:

Daily microcurrent treatment reduces sinonasal congestion over 4 weeks. Sinonasal congestion was assessed weekly using the Congestion Quanitfier 7 (CQ7) instrument. Subjects that enrolled with moderate or worse congestion (CQ7 > 15, *N* = **24**) exhibited significant reductions in congestion symptoms, compared to pretreatment, at all time points measured (a). Mean difference in CQ7 score from before treatment peaked at -8.8 (− 45.0%) points at week four (b). Data represented as mean ± SEM. ****p < 0.0001, paired, two-sided t-test

Table 2 contained incorrect data. The correct data is shown below:

For *N* = 24 subjects with CQ7 > 15 below are the corrected results:

Mean CQ7 Score: Enrollment, 19.8; Week 1, 15.4; Week 2, 13.7; Week 3, 12.4; Week 4, 11.0

Difference in CQ7 from Enrollment: Week 1, -4.4 (CI95 -2.6 to -6.2); Week 2, -6.3 (CI95 -4.2 to -8.4); Week 3, -7.4 (CI95 -4.8 to -10.1); Week 4, -8.8 (CI95 -6.5 to -11.1)

Percentage Decrease in CQ7 from Enrollment: Week 1, -23.0%; Week 2, -32.8%; Week 3, -38.1%; Week 4, -45.0%

Statistics remain unchanged at *P* < 0.0001 for all data points.
