# Supplementary material for: Prospective trial examining safety and efficacy of microcurrent stimulation for the treatment of sinus pain and congestion
Source: Bioelectron Med. 2019 Nov 20;5:18. doi: 10.1186/s42234-019-0035-x (PMC7098235; doi:10.1186/s42234-019-0035-x)

Supplementary Figure 1

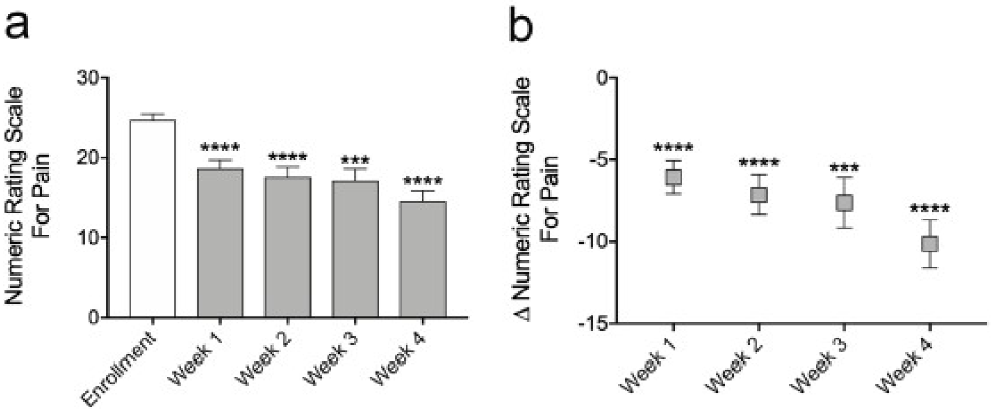

Supplement: Supplementary file 1 [file 42234_2019_35_MOESM1_ESM.docx]
